# Early clinical outcomes of simple pannus removal for mechanical aortic valve stenosis

**DOI:** 10.1186/s13019-019-1022-8

**Published:** 2019-11-27

**Authors:** Huimin Cui, Lin Zhang, Shixiong Wei, Shengli Jiang

**Affiliations:** Department of Cardiovascular Surgery, Chinese PLA General Hospital, 28 Fuxing Road, Beijing, 100853 China

**Keywords:** Mechanical aortic valve, Pannus, Prosthesis, Reoperation

## Abstract

**Background:**

This study aimed to confirm the safety and feasibility of simple pannus removal in patients with mechanical aortic valve dysfunction for pannus overgrowth by evaluating its early clinical outcomes.

**Methods:**

From March 2015 to April 2019, 24 consecutive patients with mechanical aortic valve dysfunction due to subaortic pannus underwent reoperation. In 12 patients the repeat aortic valve replacement (AVR) was performed, and 12 received the simple pannus removal to preserve the previously implanted prosthesis.

**Results:**

There was only 1 in-hospital death in simple pannus removal group. Significant differences were obtained between procedures in cardiopulmonary bypass (CPB) and aortic cross-clamp time (128.7 vs 179.7 and 74.2 vs 132.7 mins, respectively, *P* < 0.05). The C-reactive protein (CRP) in simple pannus removal group was lower on the first day (0.13 ± 0.09 vs 0.31 ± 0.22 mg/dl, P < 0.05) and continued to be lower within 1 week after operation. There was no significant difference between procedures in aortic transvalvular peak velocity and transvalvular mean pressure gradient (TMPG) (2.6 ± 0.4 vs 2.5 ± 0.4 m/s and 13.2 ± 3.6 vs 11.6 ± 2.6 mmHg, respectively, *P* > 0.05) in echocardiography 1 week after operation. In addition, the aortic transvalvular peak velocity and TMPG in echocardiography 1 week after operation in pannus removal group between the repeat and initial surgery were not statistically significant (2.6 ± 0.4 vs. 2.5 ± 0.3 m/s, 13.2 ± 3.6 vs. 13.0 ± 3.5 mmHg, *P* > 0.05).

**Conclusions:**

Simple pannus removal was a safe and effective procedure with satisfied early clinical outcomes for pannus overgrowth in mechanical aortic valve. However, further randomized and long-term follow-up studies were warranted to determine the clinical effects of the simple aortic pannus removal.

## Background

Valve thrombosis, pannus overgrowth, endocarditis, paravalvular leakage and structural valve deterioration are the most common causes of prosthetic heart valve dysfunction, among which pannus overgrowth is a rare but serious complication with poorer prognosis and higher mortality, and it has been identified as the second cause of reoperation for mechanical valve dysfunction except for thrombosis [1]. The incidence varies by types of valve prosthesis, in literature, acute dysfunction of mechanical aortic valve prostheses due to pannus is 0.73–2.4% [2]. Ellensen et al. reported 27 patients with acute valve dysfunction caused by pannus obstruction, with one-quarter of individuals dying before surgical intervention could be accomplished and 2 dying during the reoperative procedure, for an overall mortality rate of 33%, to alert us the serious nature of this complication [3]. Although this phenomenon has attracted widespread attention in the world, there is still a lack of consensus on its optimal treatment. At present, most cardiac surgeons adopt repeat valve replacement, but the high complexity of the surgery itself and the complications caused by prolonged CPB can not be ignored.

In this study, patients hospitalized for mechanical aortic valve dysfunction due to pannus overgrowth were divided into two groups. One group underwent traditional repeat AVR, while the other received simple pannus removal to preserve the previously implanted prosthesis. All operations were completed by the same experienced surgeon. We observed and compared the baseline data, surgical strategy and early outcomes of the two groups, and followed them up in order to compare the two different surgical interventions to evaluate the safety and feasibility of simple pannus removal and to determine its clinical implications.

## Methods

### Patient characteristics

From March 2015 to April 2019, 24 consecutive patients with mechanical aortic valve dysfunction due to pannus formation underwent reoperation. There were 8 men (33.3%) and 16 women (66.7%), with a mean age of 56.0 ± 6.7 (44–71) years. The mean interval between re-admission and the initial valve replacement was 14.9 ± 5.4 (1.5–23.0) years. Most of the them presented with dyspnea, lower extremity edema, syncope, dizziness, fatigue, palpitation or other cardiac insufficiency symptoms. Echocardiography showed simple aortic valve stenosis in 21 cases (87.5%), and the other 3 (12.5%) were present with stenosis combined with regurgitation. Concomitant procedures for the initial operation included mitral valve replacement (MVR) (*n* = 17), tricuspid plasty (TVP) (*n* = 4), and atrial fibrillation radiofrequency ablation (AFRA) (n = 1). All initial implanted prosthetic valves were St.Jude mechanical bileaflet valves except 2 cases of monoleaflet (1 each in aortic position and mitral position).

There were 12 patients in each group, of which 9 successively cases of repeat AVR had been completed from March 2015 to June 2017, and since then, simple pannus removal had been initiated and preferred in this patient cohort. There was no significant difference in sex, age, NYHA grade, distribution of comorbidity, interval from initial valve replacement, left ventricular ejection fraction (LVEF), left ventricular end-diastolic diameter (LVEDD), and detection of serological inflammation indicators between the two groups preoperatively. Clinical characteristics of the patients were present in Table 1. Table 2 showed the differences in hemodynamic parameters in this patient cohort, as aortic transvalvular peak velocity was 4.6 ± 0.6 and 3.9 ± 0.5 m/s, and TMPG was 45.2 ± 13.7 and 32.0 ± 9.1 mmHg in simple pannus removal group and repeat AVR group, respectively, (*P* < 0.05).

**Table 1:** Preoperative characteristics between procedures

Variable	Pannus removal	Valve re-replacement	*P*
	(*n* = 12)	(*n* = 12)	
Male/Female	3/9	5/7	0.576
Age(years)	54.9 ± 8.2	57.5 ± 7.3	0.388
BMI(Kg/m^2^)	24.4 ± 4.0	24.9 ± 3.8	0.822
Hypertension	2	2	–
Diabetes mellitus	3	2	0.404
Impaired renal function	1	1	–
Cerebrovascular disease	0	2	–
Atrial fibrillation	9(75.0%)	8(66.7%)	–
Pulmonary hypertension	1	1	–
Smoking	2	3	0.470
NYHA classes grade			
NYHA classes I	0	0	–
NYHA classes II	3(25.0%)	5(41.7%)	0.667
NYHA classes III	3(25.0%)	5(41.7%)	0.667
NYHA classes IV	6(50.0%)	2(16.7%)	0.193
interval between re-admission and initial valve replacement	15.1 ± 6.1	14.7 ± 5.1	0.898
History of cardiovascular intervention			
MVR	10(83.3%)	7(58.3%)	0.371
TVP	2(16.7%)	2(16.7%)	–
AFRA	1(8.3%)	0	–
Mean aortic valve size (mm)	20.33 ± 1.30	20.33 ± 0.98	–
Euro SCORE score	8.9 ± 7.8	8.6 ± 6.1	0.844
Serological inflammation indicators			
Hemoglobin (g/L)	107.8 ± 25.7	114.8 ± 24.3	0.528
WBC (10^9/L)	5.1 ± 1.7	5.6 ± 1.8	0.825
CPR (mg/dl)	3.1 ± 3.0	3.3 ± 2.	0.940
Total bilirubin (umol/L)	30.3 ± 36.3	19.3 ± 10.4	0.378
ALT (U/L)	17.8 ± 10.6	19.1 ± 9.9	0.484
cTnT (ug/L)	0.54 ± 0.55	0.66 ± 0.42	0.527
CKMB (ng/ml)	18.6 ± 13.6	11.4 ± 9.7	0.763
Urea (umol/L)	320.8 ± 72.3	292.6 ± 66.2	0.752
Creatinine (umol/L)	74.73 ± 17.2	73.67 ± 19.1	0.826

**Table 2:** Preoperative LV function between procedures

Variable	Pannus removal	Valve re-replacement	*P*
	(*n* = 12)	(*n* = 12)	
Aortic transvalvular peak velocity (m/s)	4.6 ± 0.6	3.9 ± 0.5	0.011
Aortic transvalvular peak pressure gradient (mmHg)	84.8 ± 24.9	60.9 ± 16.9	0.012
Aortic transvalvular mean velocity (m/s)	3.0 ± 0.5	2.6 ± 0.4	0.033
Aortic TMPG (mmHg)	45.2 ± 13.7	32.0 ± 9.1	0.011
LVEDD (mm)	4.1 ± 0.6	4.4 ± 0.8	0.366
LVEF(%)	69.7 ± 12.6	63.5 ± 8.9	0.248
Natural Mitral disease			
Severe stenosis	0	1	–
Severe regurgitation	1	0	–
Moderate regurgitation with left ventricular outflow tract obstruction	0	1	–
Mechanical mitral valve disease			
Thrombosis combined with pannus	1	0	–
Mitral TMPG ≥10 mmHg	1	1	–
Tricuspid regurgitation grade			
III	0	4(33.3%)	–
IV	8(66.7%)	5(25.7%)	0.413
Aorta dilatation	1(8.3%)	3(25.0%)	–

### Surgical indications

Indications for reoperation were (a) severe prosthetic aortic stenosis (AS) with aortic TMPG ≥40 mmHg, (b) moderate prosthetic AS (with serial aortic TMPG increment ≥20 mmHg) at the time of tricuspid or mitral valve surgery, and (c) severely reduced leaflet opening or motion.

### Surgical technique

The reoperation was performed under intravenous combined general anesthesia. Swan-Ganz catheter and double vena cava catheter were placed in the ipsilateral internal jugular vein to monitor central venous pressure (CVP), pulmartery artery pressure (PAP), pulmonary artery wedge pressure (PAWP) and cardiac output. The operation was done via a repeat median sternotomy and moderate hypothermic CPB with cannulation of the ascending aorta and vena cava or right atrium, or femoral artery or vein.

The aortic prosthesis valve was exposed through oblique incision of ascending aorta. The simple pannus removal group was treated with complete removal and debridement of pannus overgrowth, and kept the previously implanted prosthesis. Pannus removal was initiated from the mid-portion of the upper sewing ring after division of the pannus with a scalpel. The pannus then was carefully dissected on the medial and lateral sides, and was removed with a long hemostat because of the difficulty in dissecting the lower part of the mechanical aortic valve. Finally, the edge of the pannus was cauterized with electrocoagulation. The procedural time was usually 5–10 min. While the original prosthesis valve was removed and a new prosthesis valve was reimplanted after appropriate removal of perivalvular pannus in the repeat AVR group. Subsequently, the aortic incision was sutured continuously after irrigation if the aortic prosthesis valve was confirmed to be functional and accessible to the opening and closing with a plastic valve tester. Concomitant procedures including mitral or tricuspid plasty or replacement, left ventricular outflow tract dredging or ascending aorta replacement were performed in the same way as routines.

### Follow-up

All patients were continuously monitored for the changes of C-reactive protein (CRP), white blood cell (WBC), total bilirubin, alanine aminotransferase (ALT), troponin T (cTnT), creatine kinase isoenzyme (CKMB), urea and creatinine within 1 week after operation, and were examined by echocardiography to evaluate the recovery of cardiac function 1 week after operation. All patients discharged were followed up by postoperative examination back to hospital every 0.5–1 year after operation and by telephone call or a written questionnaire. Data obtained included echocardiography, functional status, survival, and cardiac-related hospital re-admission.

### Statistical analysis

The data were analyzed by SPSS 23.0 software (SPSS, Inc., Chicage, IL, USA). Continuous variables were expressed as mean and standard deviation. Categorical variables were presented as percentages. The Student t test and variance analysis were used to compare continuous variables, and the chi-squared test and Fischer’s exact test were used for analysis of categorical variables. A significant difference was considered at *P* < 0.05.

## Results

### Operative data

There was only 1 emergency operation in pannus removal group due to cardiac arrest owing to acute obstruction of prosthetic mechanical aortic valve caused by pannus combined with thrombosis. Suitable size of prosthetic valves were recommended according to the guidelines of ACC/AHA in 2017. Concomitant procedures in pannus removal group included 7 valve replacements (3 mechanical valves in mitral position and 4 bioprosthetic in tricuspid), TVP in 6, thrombectomy in 2 including 1 in mitral position. While in repeat AVR group, there were 12 AVR, using 9 mechanical valves and 3 bioprosthetic, and concomitant procedures included 3 cases each of mechanical MVR and transaortic mitral pannus removal (TMPR), 4 TVP, 2 ascending aorta replacement, and 1 case of bioprosthetic tricuspid valve replacement (TVR), left ventricular (LV) outflow tract dredging and thrombectomy, respectively. Details of primary valve replacement and reoperation were present in Table 3. Significant differences were obtained between procedures in CPB and aortic cross-clamp time (128.7 vs 179.7 and 74.2 vs 132.7 mins, respectively, *P* < 0.05). There was no significant difference in blood products use in plasma (7.4 ± 2.7 vs 7.5 ± 2.6 U), suspended red blood cells (4.7 ± 2.0 vs 4.7 ± 2.7 U), and platelets (1.2 ± 0.4 vs 1.4 ± 0.5 U) between procedures during the operation (Table 3).

**Table 3:** Operative and postoperative data between procedures

Variable	Pannus removal	Valve re-replacement	*P*
	(*n* = 12)	(*n* = 12)	
Mean CPB time (min)	128.7 ± 34.1	179.7 ± 49.9	0.005
Mean aortic cross-clamp time (min)	74.2 ± 27.9	132.7 ± 43.4	0.001
Initial procedures/Reoperation concomitant procedures			
AVR	MVR + TVP (*n* = 1) TVP (*n* = 1)	Thrombectomy (*n* = 1) MVR + left ventricular outflow tract dredging (*n* = 1) MVR + left atrium folding + TVP (*n* = 1) ascending aorta replacement (*n* = 2)	–
DVR	MVR+ mitral thrombectomy (*n* = 1) MVR + TVP (*n* = 1) thrombectomy+TVP (*n* = 1) TVR (*n* = 2) TVP (*n* = 2)	TVR (*n* = 1) TVR+ TMPR (*n* = 1) TMPR (*n* = 1) TVP+ TMPR (*n* = 1) TVP (*n* = 1)	–
DVR + TVP	TVR (*n* = 1) thrombectomy + TVP (*n* = 1)	TVR (*n* = 1) MVR + TVP (*n* = 1)	–
DVR + AFRA	TVR (*n* = 1)	–	–
Mean aortic valve size (mm)	20.33 ± 1.30	21.83 ± 1.03	–
Blood products use			
Suspended red blood cells	4.7 ± 2.0	4.7 ± 2.7	0.963
Plasma	7.4 ± 2.7	7.5 ± 2.6	0.945
Platelets	1.2 ± 0.4	1.4 ± 0.5	0.362
Mechanical ventilation time (h)	17.22 ± 6.33	25.04 ± 21.27	0.249
Postoperative complication			
Cerebral infarction	1	0	–
Permanent pacemaker implantatoion	0	1	–
Infective shock	0	1	–
Pleural effusion	0	2	–
Valve hemodynamics one week after operation			
Aortic transvalvular peak velocity (m/s)	2.6 ± 0.4	2.5 ± 0.4	0.431
Aortic transvalvular peak pressure gradient (mmHg)	28.6 ± 8.4	25.6 ± 7.4	0.332
Aortic transvalvular mean velocity (m/s)	1.5 ± 0.4	1.5 ± 0.2	0.215
Aortic TMPG (mmHg)	13.2 ± 3.6	11.6 ± 2.6	0.174
LVEF(%)	62.4 ± 7.4	59.6 ± 10.5	0.615
ICU stay (d)	7.5 ± 5.21	8.75 ± 7.26	0.654
In-hospital death	1	0	–
Hospital stay (d)	33.3 ± 10.7	48.8 ± 28.2	0.103
NYHA classes grade during this follow-up interval			
NYHA classes I	7(63.6%)	7(58.3%)	–
NYHA classes II	4(36.4%)	3(25.0%)	–
NYHA classes III	0	1(8.3%)	–
NYHA classes IV	0	1(8.3%)	–

**Fig. 1:**
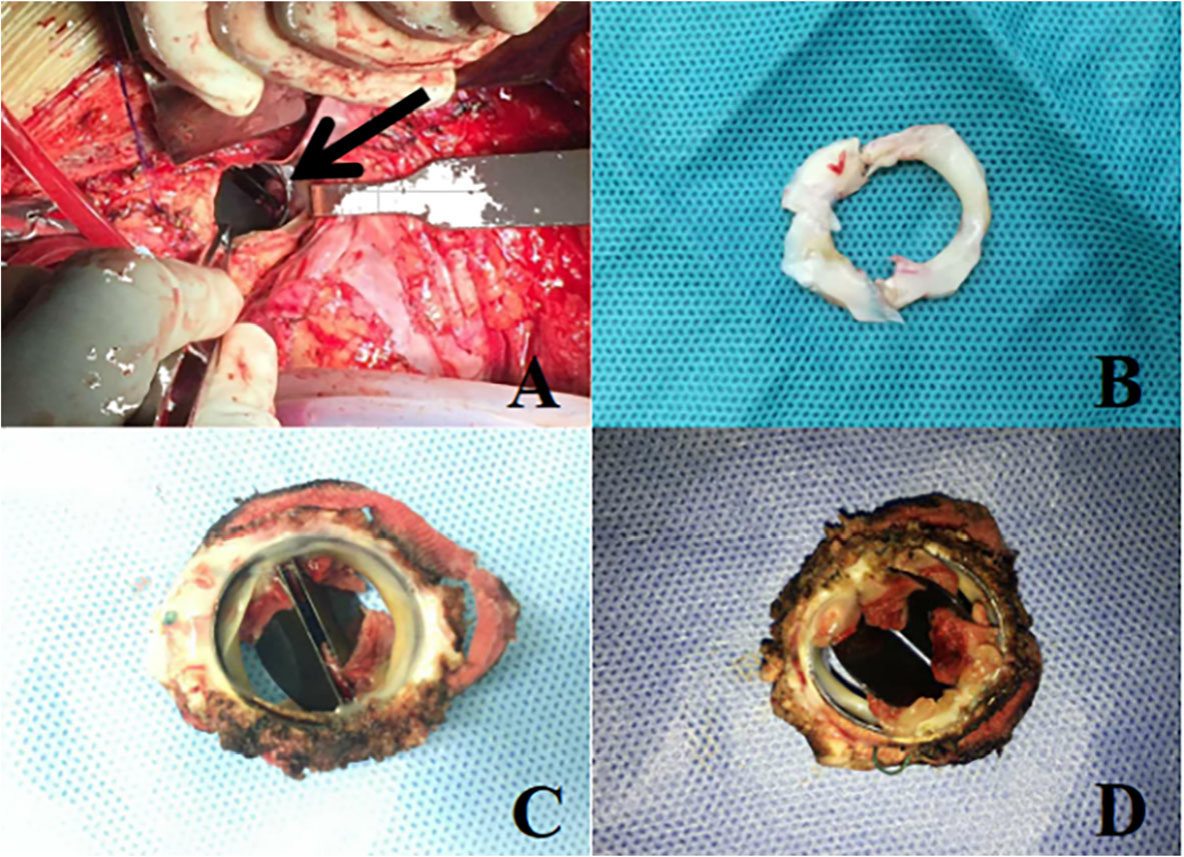
Operative findings at redo surgery. **a** A relatively fresh thrombus laid between the pannus and the leaflet of the prosthesis (arrow); **b** Excised pannus from ventricular side; **c**, **d** A concentric pannus formation was seen from arterial and ventricular side

All 12 removed mechanical aortic valves were macroscopically examined after operation, and subaortic pannus was identified in all patients, of which severe subvalvular pannus overgrowth was detected in 10 cases (83.3%) (Fig. 1). The proportion of overgrowth from the annulus was almost even and a subvalvular membrane was created by pannus overgrowth from the ventricular side, which restrict the effective orifice area (EOA) especially for each commissure. Pannus arises from ventricular aspect of the prosthesis encroaching its leaflets causing stenosis or it may remain localized causing left ventricular outflow tract obstruction without affecting valve function.

Pathological findings suggested that pannus was composed of proliferative fibrous tissue with hyaline degeneration and local mucinous degeneration, and a little chronic inflammatory cell infiltration could be seen.

### Early results

**Fig. 2:**
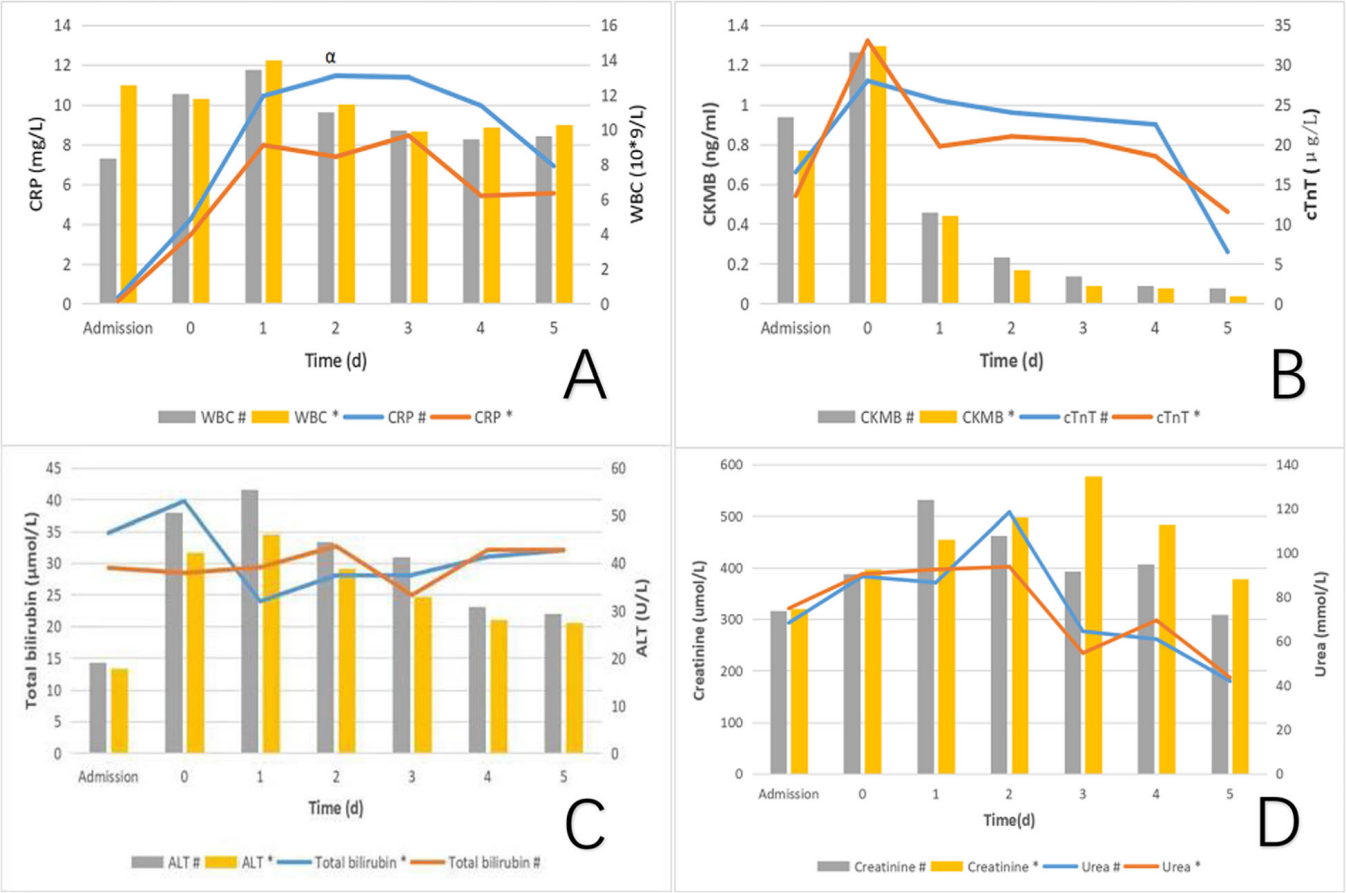
Trends of CRP and WBC (**a**), ALT and Total bilirubin (**b**), cTnT and CKMB (**c**), Urea and Creatinine (**d**) within 1 week after operation between procedures. # In repeat AVR group, *In smiple pannus removal group, α: *P* < 0.05

Global systolic function improved in all patients, as in prosthetic valve aortic transvalvular peak velocity (2.6 ± 0.4 vs 4.2 ± 0.7 m/s), transvalvular mean velocity (1.5 ± 0.3 vs 2.7 ± 0.4 m/s), transvalvular peak pressure gradient (27.3 ± 7.7 vs 70.8 ± 24.2 mmHg), and TMPG (12.6 ± 3.2 vs 36.8 ± 12.0 mmHg) were all significantly decreased (*P* < 0.05) to normal level in echocardiography 1 week after operation. However, there was no significant difference (*P* > 0.05) in valve hemodynamics in aortic transvalvular peak velocity (2.6 ± 0.4 vs 2.5 ± 0.4 m/s), transvalvular mean velocity (1.5 ± 0.4 vs 1.5 ± 0.2 m/s), transvalvular peak pressure gradient (28.6 ± 8.4 vs 25.6 ± 7.4 mmHg), TMPG (13.2 ± 3.6 vs 11.6 ± 2.6 mmHg) and LVEF (62.4 ± 7.4 vs 59.6 ± 10.5%) between procedures. In addition, the aortic transvalvular peak velocity and TMPG in echocardiography 1 week after operation in pannus removal group between the repeat and initial surgery were not statistically significant (2.6 ± 0.4 vs. 2.5 ± 0.3 m/s, 13.2 ± 3.6 vs. 13.0 ± 3.5 mmHg, *p* > 0.05). The systemic inflammatory response index of CRP was lower on the first day (0.13 ± 0.09 vs 0.31 ± 0.22 mg/dl, *P* < 0.05) and continued to be lower within 1 week after operation in simple pannus removal group. However, other serological inflammation indicators including WBC, total bilirubin, ALT, cTnT, CKMB, urea and creatinine were failed to find significant difference between procedures (Fig. 2). In addition, the length of postoperative mechanical ventilation (17.22 ± 6.33 vs 25.04 ± 21.27 h), stay in ICU (7.50 ± 5.21 vs 8.75 ± 7.26 d) and hospitalization (33.3 ± 10.7 vs 48.8 ± 28.2 d) were not statistically significant (*P* > 0.05) between procedures.

There was only 1 in-hospital death in simple pannus removal group. This patient could not be revived after positively dehydration and lowering intracranial pressure treatment for a large area new cerebral infarction, and remained vegetative state until death on the 46th day after operation. Early complications included 1 permanent pacemaker implantation for high atrioventricular block and 1 infective shock due to *Klebsiella pneumoniae* infection in repeat AVR group. Both of them and the emergency case were cured and discharged uneventfully.

### Follow-up

All patients discharged were successfully followed up until July 2019 and mean follow-up was 24.4 ± 12.2 (3–50) months. Functional classification improved, as NYHA status changed from the preoperative mean of 3.00 to 1.52 during this follow-up interval. 13 of 15 surviving patients who were in NYHA class III/IV preoperatively improved to class I/II, with no difference between procedures (8/8 vs 5/7, *P* > 0.05). Among the 2 residual cases in repeat AVR group, one suffered from decreased physical activity due to renal insufficiency and the other presented lower extremity edema after fatigue. Up to now, all patients showed good cardiac function by echocardiography with satisfied prosthetic valve anatomy and hemodynamics.

## Discussion

Prosthetic valve dysfunction was one of the most serious complications after mechanical valve replacement and the optimal management remained controversial. In literature, the incidence was 0.1–6.0% per patient year and the mean interval between the initial valve replacement and reoperation was 10–16 years [4]. Prosthetic valve dysfunction at aortic position is commonly caused by pannus formation which is an uncommon and lifethreatening complication. Its incidence varies between 1.8% in tilting disc to 0.73% in bileaflet valves [2]. Previous studies had shown an increasing occurrence of pannus formation with time after implantation of any mechanical valve and it was known to occur more quickly and common in the aortic position than in the mitral position (70 vs 21%) because of the smaller size of the aortic valve [1, 2, 5]. As pannus grows from the periannular to the prosthetic valve ring and disc, it may inhibit opening or closure of the valve disc, thereby leading to acute aortic obstruction or regurgitation, the presentation can vary from asymptomatic to exertional dyspnea or decreased exercise tolerance to angina, syncope until heart failure (HF) or even sudden cardiac arrest. In our series, the interval between the initial valve replacement and reoperation was 14.5 ± 5.4 years, and most of the patients on re-admission were present with cardiac insufficiency symptoms such as dyspnea (66.7%), fatigue (29.2%) and lower extremity edema (25.0%), including 1 cardiac arrest.

Pannus was suspected in patients who exhibited high gradients on echocardiography. The diagnosis of pannus could be combined with clinical suspicion, Doppler examination by measuring hemodynamic parameters, transthoracic echocardiography (TTE) to rule out structural failure and patient-prosthesis mismatch (PPM), and transesophageal echocardiography (TEE) to determine certain characteristics differentiating from thrombosis such as preserved prosthetic disc motion and evidence of a hyper-reflective mass of decreased length and motion [5]. Barbetseas et al. evaluated the sensitivity of TEE in diagnosing prosthesis dysfunction at 83% [5]. In our study, the sensitivity of TEE to pannus diagnosis was up to 91.7%, but the specificity was only 29.2%. Accurate diagnosis of this disorder had become of utmost importance, since different etiology of valve obstruction had different strategy, and patients with valve thrombosis could benefit from the use of thrombolytics. Recently, despite advancement in multislice CT and 3D TEE facilitated more precise visualization and quantification of the shape and extent of the pannus, the relationship between prosthetic valve dysfunction and the extent of pannus involvement remained unclear [6].

The reported risk factors for pannus formation were prosthetic valve design, biocompatibility, smaller annuli, surgical techniques, blood flow turbulence, shear stress, high transvalvular pressure gradients, young patient, female sex, rheumatic heart disease (RHD), DVR status (aortic and mitral valves), and inadequate anti-coagulation [3, 7]. In this series, most patients were women (66.7%) and had had a DVR (70.8%) because of rheumatic heart pathology (100%), which were consistent with previous literature reports. Since the design of a tilting disc valve is more prone to fibrous tissue obstruction than a bileaflet one, the 2 monoleaflet valves in this series were given repeat valve replacement. Pannus in combination with thrombosis was found in 3 patients (12.5%). Thrombosis which had been considered as a major risk factor for mechanical prosthetic valve dysfunction was usually due to inadequate anticoagulation and associated with atrial fibrillation or low cardiac function, and pannus was often seen with a superimposed thrombus, thereby enhancing the obstructing effect on the disc motion, which was also often the cause of critical status such as acute cardiogenic shock, requiring emergency surgery, as 1 proven emergency case in this series. This highlighted the need for adequate anticoagulation and regular follow-up after mechanical valve replacement.

Subvalvular pannus formation resulted in substantial changes in the transvalvular peak velocity, corresponding transvalvular pressure gradient (TPG) and opening angle of the prosthetic valve. Maximum flow velocity and corresponding TPG were mostly affected by pannus width and it had been reported that pannus formation elevated TPG to > 2.5 times higher than that without pannus formation if pannus width was 25% of the valve diameter [6]. Increased aortic TMPG was often associated with LV diastolic dysfunction, LV hypertrophy, secondaty pulmonary hypertension and a late significant TR, and was often accompanied by worsened clinical manifestations. In this series, preoperative aortic TMPG was as high as 45.2 ± 13.7 mmHg in simple pannus removal group and 32.0 ± 9.1 mmHg in repeat AVR group, and both were significantly reduced to normal level after reoperation (45.2 ± 13.7 vs 13.2 ± 3.6, and 32.0 ± 9.1 vs 11.6 ± 2.6 mmHg, respectively, *P* < 0.05), with no difference between procedures, which might imply that simple pannus removal could reach the standard of valve re-replacement. In addition, the aortic transvalvular peak velocity and TMPG in echocardiography 1 week after operation in pannus removal group between the repeat and initial surgery were not statistically significant (2.6 ± 0.4 vs. 2.5 ± 0.3 m/s, 13.2 ± 3.6 vs. 13.0 ± 3.5 mmHg, *P* > 0.05), which might indicate that simple pannus removal could get the early standard of primary valve replacement.

To avoid prolonging the natural course of the disease and its repercussion on the LV and the quality of life of affected patients, timely surgical intervention was the most effective treatment, despite controversy existed regarding the optimal management. In our series, global systolic function improved in all patients after reoperation, as aortic transvalvular peak velocity and TMPG (2.6 ± 0.4 vs 4.2 ± 0.7 m/s and 12.6 ± 3.2 vs 36.8 ± 12.0 mmHg, respectively, *P* < 0.05) were significantly decreased to normal in the echocardiography 1 week after operation. Albeit there were reports that valve function could be normal after debridement, it should be noted that the pannus generally developed on both prosthetic surfaces and adhered firmly to the prosthetic valve disc especially for each commissure, which made complete removal difficult, particularly in patients with extensive, circumferential pannus, and any slight damage of the disc surface was likely to cause thrombosis. Therefore, repeat valve replacement had been the conventional surgical treatment worldwide in mechanical valve dysfunction due to pannus formation for many years or even now. Pyo Won Park et al. showed that overall survival and event-free survival rates of repeat AVR for subaortic pannus in mechanical aortic valve at 10 years were 88 and 51%, respectively [8]. However, we believed it was enough to debride the pannus instead of prosthetic valve replacement owing to the relatively short surgical duration, and debridement may have lower risk and mortality than valve replacement, despite of 1 in-hospital death in simple pannus removal group. Moreover, by saving the original prosthetic valve, it might contribute in decreasing the possibility of paravalvular leakage and preventing an arrhythmic complication, as 1 permanent pacemaker implantation for high atrioventricular block was occurred in repeat AVR group. Furthermore, preserving the prosthesis was probably better than re-replacement in order to minimize invasion and reduce bio-reaction because re-replacement required many maneuvers around the aortic annulus and the insertion of a new mechanical prosthesis might trigger a new, more rapid phlogistic reaction in a patient with a foreign prosthesis-related inflammatory reaction already activated by the previous event. Actually, patients who underwent reoperation were often with poor conditions and worsened cardiac function, therefore, pannus removal might be considered in patients with high surgical risks, on the other hand, based on the fact that no matter what treatment would be taken, the mortality rate of prosthetic dysfunction was significantly higher than the primary valvular disease [4].

Significant differences were obtained between procedures in CPB and aortic cross-clamp time (128.7 vs 179.7 and 74.2 vs 132.7 mins, respectively, *P* < 0.05) in our study. We believed the shortening duration of CPB can significantly shorten the ischemic time, reduce blood damage, and control systemic inflammatory response. It was also noted that the systemic inflammatory response index of CRP in our series was lower on the first day (0.13 ± 0.09 vs 0.31 ± 0.22 mg/dl, *P* < 0.05) and continued to be lower within 1 week after operation in simple pannus removal group. Fortunately, several case reports and small series of patients had reported good results of pannus removal, instead of prosthesis reimplantation. Ahmad K Darwazah reported a recurrent pannus observed in a female patient who needed repeated surgical intervention to excise a localized pannus without re-replacement of a well functioning prosthetic valve [9]. Several years prior, Pyo Won Park et al. also reported 34 patients (median age, 57 years; 30 women) with rheumatic disease underwent pannus removal on the ventricular side of a mechanical mitral valve through the aortic valve during reoperation between 2004 and 2016, and concluded that TMPR was a safe and effective procedure for patients with malfunction or stenosis of a mechanical mitral valve [7]. In our series, 3 cases of TMPR were performed during repeat AVR, and all of the 3 mechanical mitral valves and the other 11 mechanical aortic valves in the simple pannus removal group were all in good status in the recent echocardiographic follow-up. As pannus formation was confirmed in all patients during reoperation, subsequently, pannus removal via aortotomy has become a routine procedure to identify significant subaortic stenosis in patients with moderate mechanical AS at the time of mitral or tricuspid valve surgery.

Hdeki et al. revealed the morphological, histological and immunohistochemical mechanism of pannus formation using resected pannus tissue from 11 patients with prosthetic valve dysfunction in the aortic position who underwent reoperation between 1980 and 1999, and demonstrated that pannus, constituted with collagen and elastic fibrous tissue accompanied by chronic inflammatory cells infiltration, appeared to originate in the neointima in the periannulus of the left ventricular septum and extend into the pivot guard, interfering with the movement of the straight edge of the leaflet, and pannus formation may be associated with a process of periannular tissue healing via the expression of transforming growth factor-beta [10]. Our results coincided with those obtained by Hdeki et al. Although the detailed mechanism of its formation has not yet been fully demonstrated, based on previous study and our research, we considered pannus was a fibroelastic hyperplasia originating from the left ventricular septum, which was composed of proliferative fibrous tissue with hyaline degeneration and local mucinous degeneration infiltrated by a little chronic inflammatory cell, and induced by protracted periannular wound healing or phagocytosis to foreign prosthesis through persistent chronic inflammatory reactions via the expression of transforming growth factor-beta, and often took longer time to become clinically manifest.

There was a concern regarding re-growth of the pannus after reoperation, because any inhibition for healing process was unfeasible during recovery from surgery. Although certain authors suggested recurrence was a finding of low prevalence and high mortality and occurred predominantly in patients who underwent pannus resection without valve replacement [11], the difference in recurrent pannus between redo valve replacement and pannus removal was not statistically significant, and all series agreed in that the time to re-intervention was prolonged. Nakatani Y et al. had administered tranilast, N-(3,4-dimethoxycinnamoyl) anthranilic acid, an anti-allergic drug with multiple effects, including the inhibition of TGF-β1 and the prevention of fibrosis in various pathophysiological settings, as an adjunctive therapy to prevent fibrous tissue overgrowth [12]. Therefore, inhibiting or controlling the local expression of TGF-beta and its receptors might become one of the future research directions in the prevention of pannus formation. The silver coating (Silzone®) of the sewing cuff of the mechanical heart valve prostheses was once designed in an effort to reduce the incidence of prosthetic valve endocarditis (PVE) and its sequelae, by St. Jude Medical Inc. (Minneapolis, MN, USA) in 1997, however, it was voluntarily recalled (January 2000) due to an unusually high incidence of paravalvular leaks [13]. Schwartz et al. hypothesized that coronary restenosis was attributable to excessive neointimal formation accompanied by myofibroblasts and inflammatory cells [14]. According to their hypothesis and the design concept of Silzone® valve, and based on the consensus that drug-eluting stents was superior to bare metal stents in reducing in-stent restenosis, basic research of prosthetic valve design and biocompatibility in drug-eluting sewing cuff might become another future research direction in the prevention of pannus formation.

## Limitations

The limitation of this study related to the fact that this was non-randomized, retrospectively analyzed data from a small number of patients with short follow-up period in a single center experience. Further randomized and long-term follow-up studies were warranted to determine the clinical effects of the simple aortic pannus removal.

## Conclusion

Simple pannus removal was a safe and effective procedure with satisfied early clinical outcomes for pannus overgrowth in mechanical aortic valve. However, further randomized and long-term follow-up studies were warranted to determine the clinical effects of the simple aortic pannus removal.

## Data Availability

Supporting data are available through the corresponding author on reasonable request.

## Abbreviations

AFRA: Atrial fibrillation radiofrequency ablation
ALT: Alanine aminotransferas
AS: Aortic stenosis
AVR: Aortic valve replacement
CKMB: Creatine kinase isoenzyme
CPB: Cardiopulmonary bypass
CRP: C-Reactive protein
CT: Computed tomography
cTnT: Troponin T
CVP: Central venous pressure
DVR: Aortic and mitral valves replacement
EOA: Effective orifice area
HF: Heart failure
LV: Left ventricular
LVEDD: Left ventricular end-diastolic diameter
LVEF: Left ventricular ejection fraction
MVR: Mitral valve replacement
PAP: Pulmartery artery pressure
PAWP: Pulmonary artery wedge pressure
PPM: Patient-prosthesis mismatch
RHD: Rheumatic heart disease
TEE: Transesophageal echocardiography
TMPG: Transvalvular mean pressure gradient
TMPR: Transaortic mitral pannus removal
TPG: Transvalvular pressure gradient
TTE: Transthoracic echocardiography
TVP: Tricuspid plasty
TVR: Tricuspid valve replacement
WBC: White blood cell
